# Supplementary figures and images for: Dynamics of G6PD activity in patients receiving weekly primaquine for therapy of Plasmodium vivax malaria
Source: PLoS Negl Trop Dis. 2021 Sep 8;15(9):e0009690. doi: 10.1371/journal.pntd.0009690 (PMC8452019; doi:10.1371/journal.pntd.0009690)

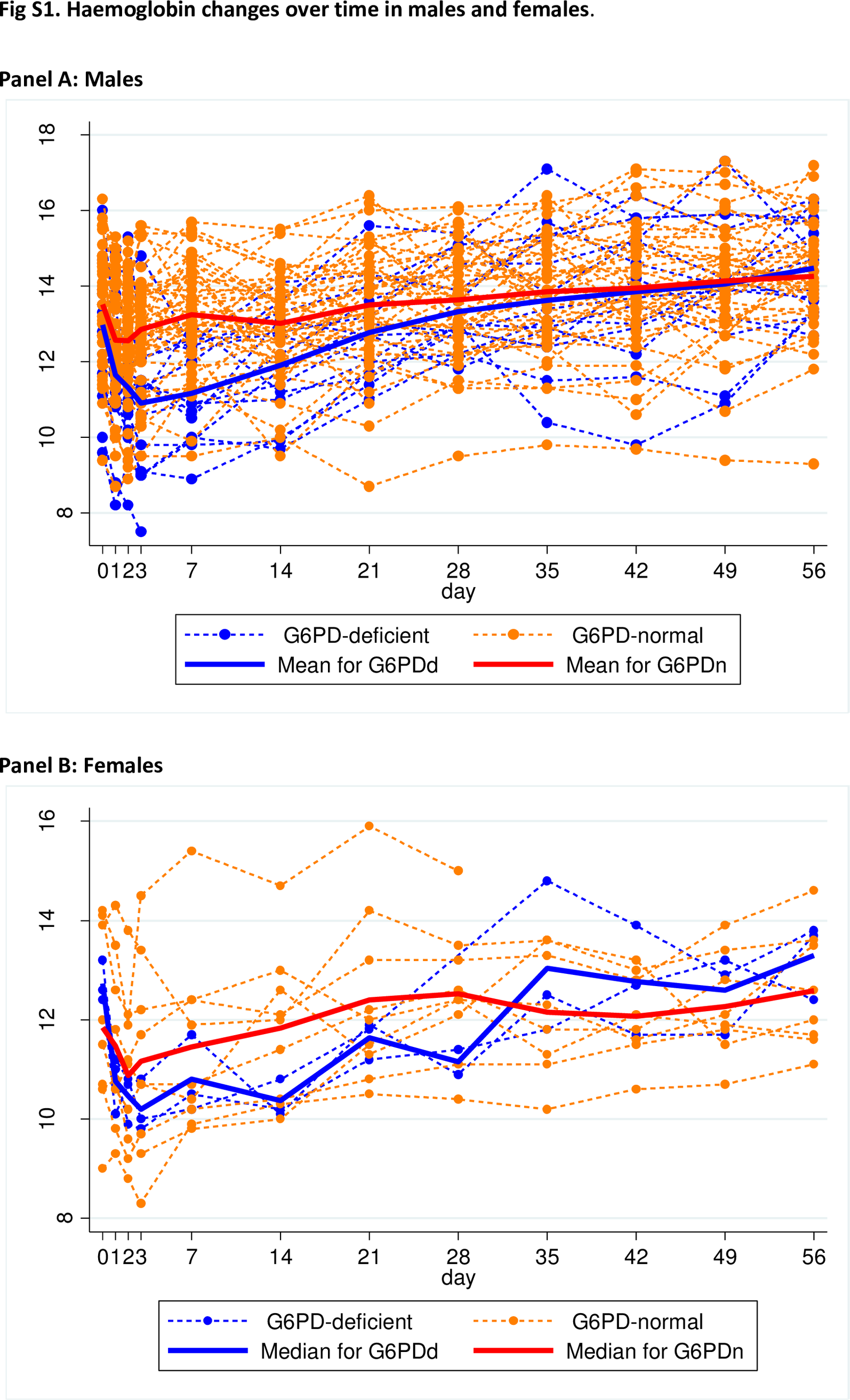

Supplement: S1 Fig — Panel A: males. Panel B: females. (TIF) [file pntd.0009690.s001.tif]

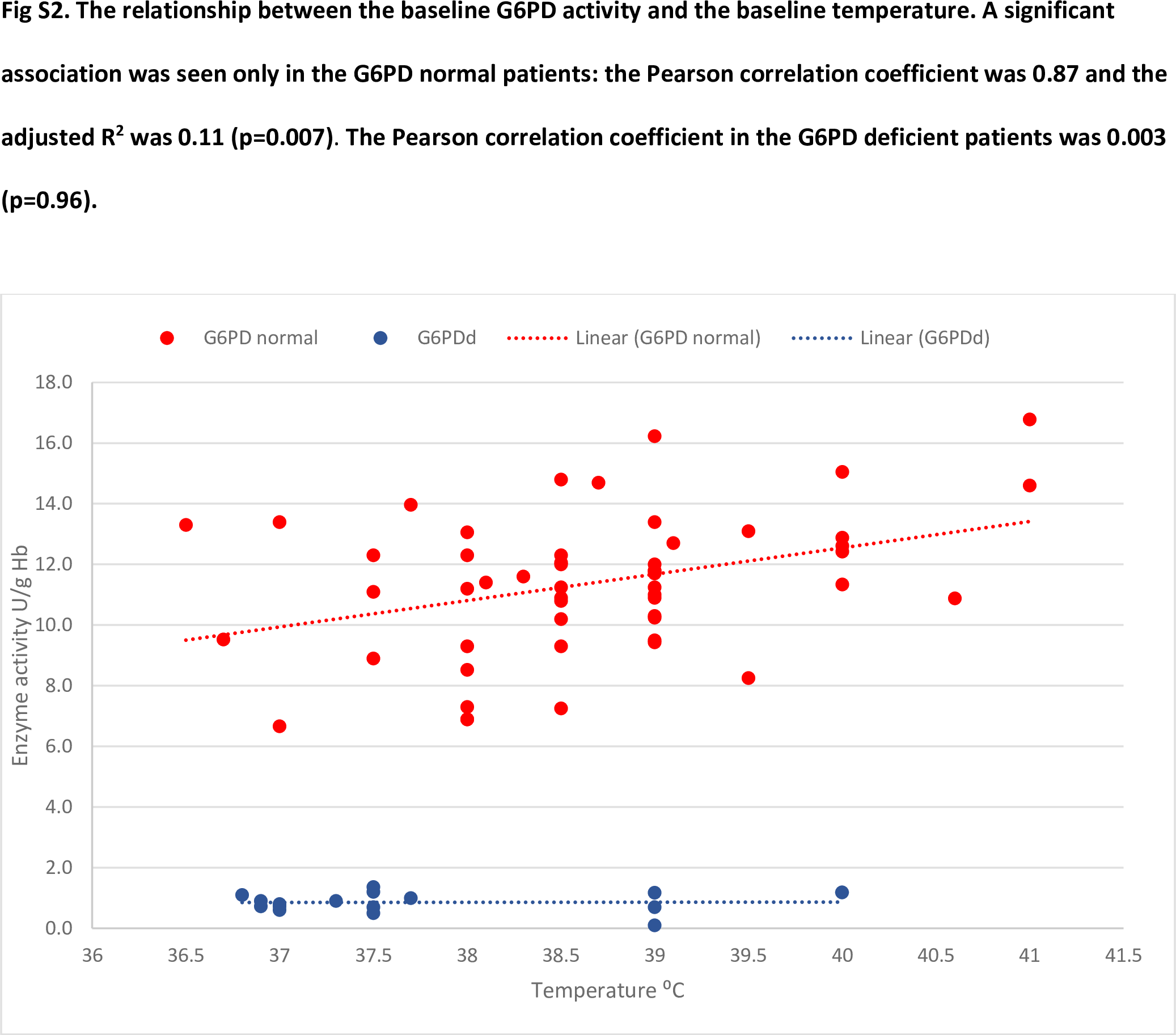

Supplement: S2 Fig — A significant association was seen only in the G6PD normal patients: the Pearson correlation coefficient was 0.87 and the adjusted R2 was 0.11 (p = 0.007). The Pearson correlation coefficient in the G6PD deficient patients was 0.003 (p = 0.96). (TIF) [file pntd.0009690.s002.tif]

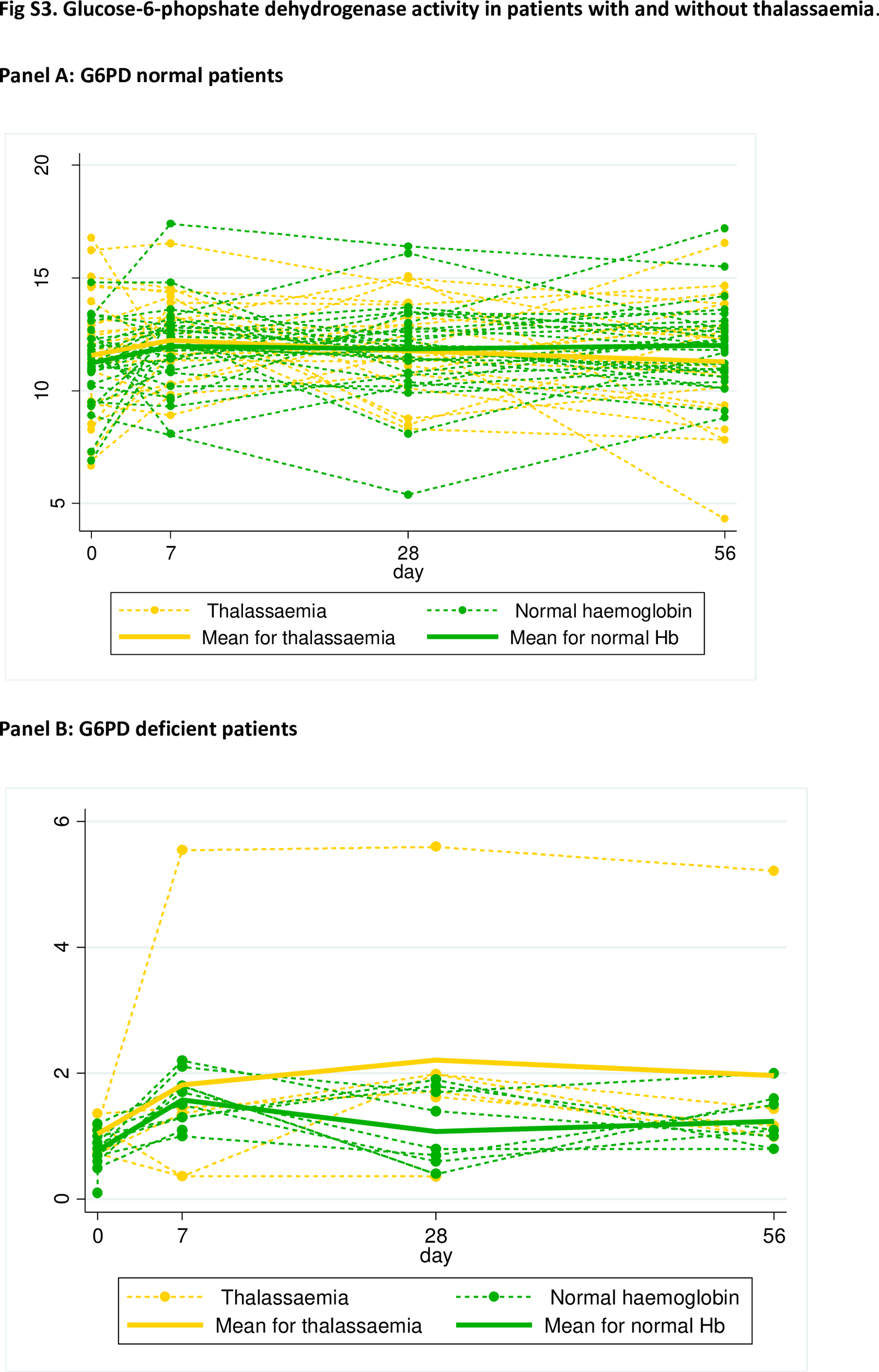

Supplement: S3 Fig — Panel A: G6PD normal patients. Panel B: G6PD deficient patients. (TIF) [file pntd.0009690.s003.tif]

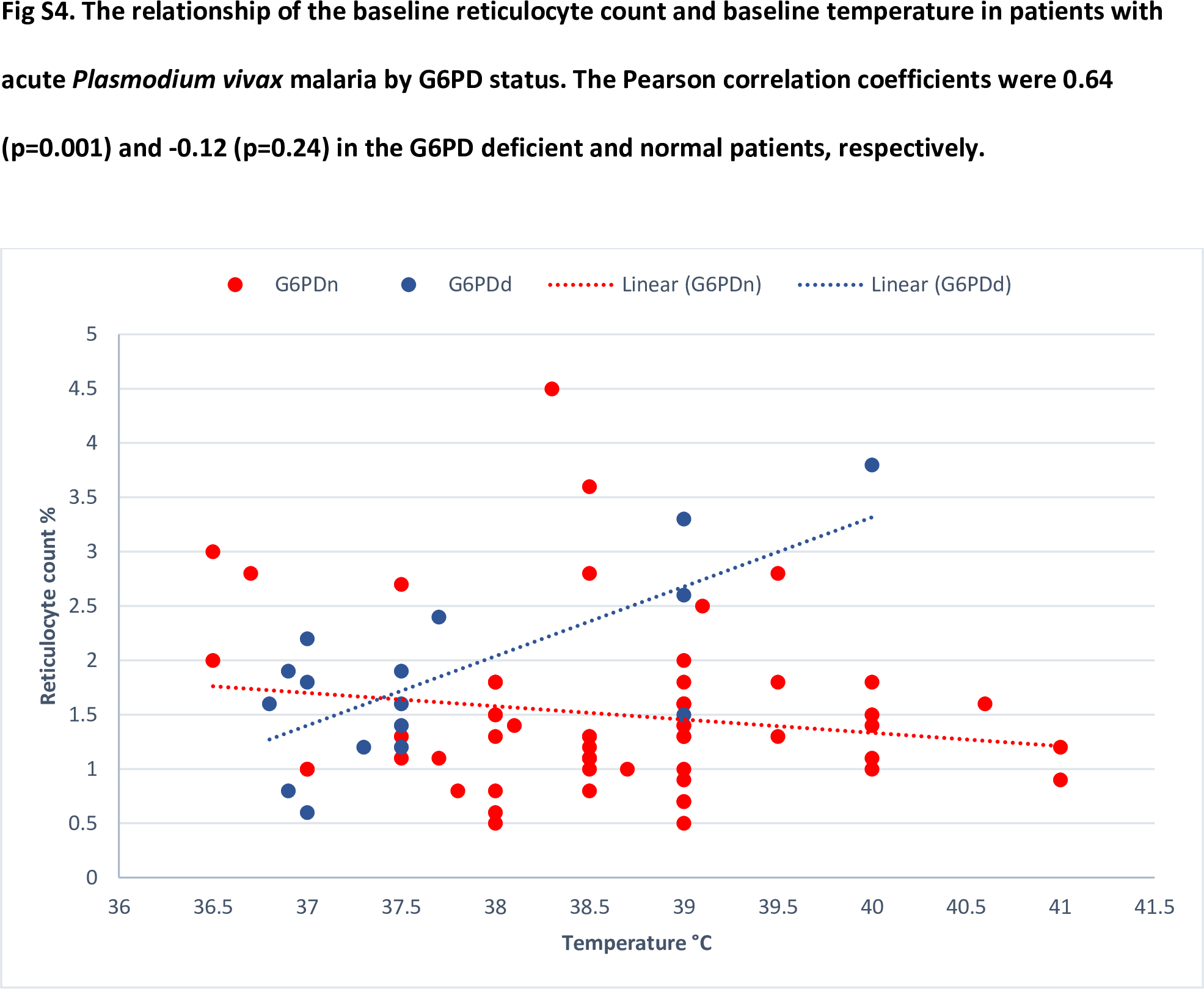

Supplement: S4 Fig — The Pearson correlation coefficients were 0.64 (p = 0.001) and -0.12 (p = 0.24) in the G6PD deficient and normal patients, respectively. (TIF) [file pntd.0009690.s004.tif]
