# Supplementary material for: Dynamics of G6PD activity in patients receiving weekly primaquine for therapy of Plasmodium vivax malaria
Source: PLoS Negl Trop Dis. 2021 Sep 8;15(9):e0009690. doi: 10.1371/journal.pntd.0009690 (PMC8452019; doi:10.1371/journal.pntd.0009690)
Supplement: Attachment — Submitted filename: response to reviewers-G6PD_v0.5.docx [file pntd.0009690.s006.docx]

**6^th^ July 2021**

***Re:*** Dynamics of G6PD activity in patients receiving weekly primaquine for therapy of *Plasmodium vivax* malaria

Dear Dr. Monteiro & Lopez-Perez,

Thank you for passing on the detailed and useful comments of the reviewers. Herein, we respond.

Yours sincerely,

Bob Taylor.

Reviewer #1: The objectives of the study are not as clearly stated as desired. It is assumed (but unclear) that the primary objective is hypothesis-testing for the FST diagnosis question, but the authors do not explicitly state this.

If the primary objective is hypothesis-testing, the study does not seem adequately powered and the sample population not large enough. That is, the authors claim that the FST qualitative test accurately diagnoses individuals with G6PD Viangchan but this conclusion is based on only 15 geno- and phenotypically confirmed G6PDd males and 3 heterozygous female patients. Further, the authors need to provide a justification for the sample size. The authors should consider whether or not it is even appropriate to test this hypothesis for inclusion in this manuscript.

Regarding the reticulocyte portion of the study, as this is an exploratory analysis, the sample size is acceptable.

Response

The study is not testing a formal hypothesis. The data we present are from a small safety study of weekly primaquine and wish to document the variations in G6PD activity and reticulocyte count at baseline and following treatment.

Therefore, this is a descriptive study with exploratory analyses and without a predetermined sample size.

We have clarified this in the paper (page 7, lines 110-112).

Additionally, while the authors stated which statistical methods they employed, explicit mention of the assumptions tested (and how) is requested, as well as mentioning in the figure legends which statistical test was performed and for which variables the parametric assumptions were not met.

We have rewritten the analysis section and simplified some of the analyses (see page 8).

Proportional data between groups were compared using chi squared or Fisher’s exact test, as appropriate. Normally distributed data were analysed by paired (within groups) or unpaired ‘t’ (between groups) tests and skewed data by the corresponding non-parametric tests. Data distribution was assessed using the sktest command in Stata. The sensitivity and specificity of the FST to diagnose G6PDd patients were calculated against the G6PD genotype as the “gold standard.” Using backward stepwise multivariate regression, we examined the effects of age, sex, illness days, thalassaemia, and baseline values of temperature, parasitaemia, haemoglobin and reticulocyte count on the mean baseline G6PD activity. Linear mixed effects modelling was used to assess the independent effects of age, sex, illness days, baseline splenomegaly, thalassaemia, baseline reticulocyte count and parasitaemia, the fall in Hb (baseline – nadir Hb) and G6PD activity over time. The relationship between two continuous variables was determined by simple linear regression to determine the Pearson correlation coefficient.

Finally, critical detail on the regression model building is requested; specifically, which variables were included in the initial full model, what was the process of elimination, what was the P-value cutoff for elimination and re-entry, were there any confounding variables and if so how was it dealt with

Whist it is conventional to include, in a regression model, variables that have been identified in a univariate analysis, here we decided to include clinically relevant variables from the start using a backward step approach (page 8).

Reviewer #2: The objectives were not clear:
1. In both the abstract and introduction, the authors state that one of the goals of the study is to “assess the impact of acute vivax malaria and of its treatment on G6PD activity levels” however, to assess the impact of vivax malaria on G6PD activity, don’t you need an uninfected comparison group? I didn’t see any data on the impact of malaria on G6PD in this study, so perhaps it should be added or this goal removed.

Ideally, yes but this requires another study with matched controls of G6PD normal and deficient healthy volunteers who are given DHA piperaquine and weekly primaquine.

Our paper is a spin off descriptive paper arising from a small clinical trial that assessed the safety of weekly primaquine in G6PD deficient and normal patients. Thus, our patient population is fixed for this descriptive manuscript. We have clarified this in the paper (page 7, lines 110-112).

2. Since you are assessing the performance of a diagnostic test in the setting of an active malaria infection, would it be helpful to use ROC curves to assess the performance of the test? Sensitivity and specificity?

***We have now added in diagnostic test parameters in the paper - sensitivity and specificity, cognizant that our sample size is small.***

A ROC would turn the analysis on its head i.e. at what level of enzyme activity would we expect the FST to be positive or negative. In clinical practice, it is the FST result that is important and we have assessed it against the G6PD genotype.

3. Would it be helpful to compare the performance of the test in uninfected individuals to see if it is changed by malaria infection?

Yes but our study was in patients. In retrospect, we could have assessed the FST during the convalescence. There are several large studies that have assessed the performance of the FST in patients and healthy individuals and it performs very well in both settings. We have referenced several papers in the manuscript.

4. To what extent was drug treatment versus the disease itself driving the changes in G6PD activity observed over time? Is there data available for patients who did not receive primaquine?

No unfortunately not. This is limitation of the study. A no primaquine arm would have been very useful and given us data on G6PD activity in the absence of an oxidant effect of primaquine. It is worth pointing out that it was very challenging to find G6PD deficient vivax-infected patients, a challenge also experienced by others in Cambodia (Dysoley et al) and elsewhere (Taylor et al, 2019).

5. I’m surprised that G6PD activity has to be adjusted for MCV. I would expect its concentration in the cytoplasm to be the same, even if the RBC is smaller, and therefore its concentration relative to Hb would be unchanged, even if the activity per RBC is lower.

Previous data show (Piomelli et al, cited in the paper) that, in thalassaemia, the G6PD activity per red cell was similar in thalassaemic and normal individuals but, because of the microcytosis, the G6PD activity per gram of haemoglobin was increased in thalassaemia trait.

Therefore, in our paper, we adjusted for this using the formula: mean MCV_thalassaemia_/mean MCV_normal Hb_ . This was done for each time interval that we had MCV data. The mean correction was 0.9 i.e. the adjusted G6PD activity in those patients with thalassaemia/HbE was reduced by a mean of 10%. This resulted in a small reduction of G6PD activity.

Reviewer #3: Methods are sound

--------------------

Reviewer #1: Though it is not immediately clear to this reviewer and required close scrutiny of the text and table/figure legends, the authors appear to have followed their analysis plan. However, the order of results is somewhat confusing, as the authors flip-flop the order from the Introduction. That is, in the Intro the authors first discuss the performance of the FST then the reticulocyte/G6PD activity studies but then in the Results they present the retic/G6PD activity first and FST performance second--this should be corrected.

We have now matched the Introduction and the Results so that the order of topics is the same.

Furthermore, figures and tables require revision. First, none of the figures have titles on the y-axis; each graph should be able to stand alone.

We doubled checked the figures and all of them were labelled properly. Perhaps, there was a technical issue. All Figures are labelled.

Second, this reviewer requests "A" and "B" designations for two-panel figures.

Yes, we have done this.

Third, it is requested that all correlation coefficients regardless of statistical significance be presented in the manuscript.

Yes, this has been done.

Fourth, it is not clear what is meant by the bolded outcome variables in Table 2--what exactly
does the variable Reticulocyte Dynamics mean, is it continuous or categorical, etc.

Reticulocyte dynamics means the changes in reticulocyte counts over time so is a continuous variable. For clarity we have used the latter label. Table 2 has been amended. We removed the first row and put those data in the text.

In general, it is preferred to see the marker of central tendency as median with 95% CI if the authors are concerned that their population is not normally distributed or large enough to assume normality (which it is not).

We have used mean for some parameters to match the multivariable regression analyses. Whilst we agree that central tendency is important, showing the full range of clinical variables is more informative. Accordingly, we have not shown the 95% CIs but refer to show the ranges.

Reviewer #2: 6. Table 2 is unclear, and I could not find a definition for the factor “Reticulocyte dynamics” or the outcome variable “G6PD activity dynamics” or the outcome variable “Reticulocyte dynamics”.

We have dropped the terms “Reticulocyte and G6PD activity dynamics.”

Reviewer #3: Yes, except spaghetti plots are hard to look at.

Whilst we sympathise with this reviewer, we prefer to keep the spaghetti plots as they give us a better idea of the individual changes of key clinical parameters.
--------------------


Reviewer #1: For the FST performance, this reviewer is concerned that the authors are over-generalizing their conclusion based on limited sample size. Furthermore, the limitations section of this study requires additional limitations and more extensive discussion. Here it may be prudent to discuss published sensitivity and specificity of the FST, whether there are known correlations between quantitative and qualitative testing (that is, at what quantitative threshold of enzyme activity does the FST correctly diagnose a patient). Discussion on this second point would be helpful for framing the FST performance results in the context of a small sample size.

We have toned down the interpretation of the FST results and expanded the Limitations section. We have also brought out more the literature on FST and included data from qualitative rapid diagnostic tests (see pages 16-17, lines 270-277).

Reviewer #2: 7. In the discussion, you refer to the safety and tolerability of the primaquine course and refer to one patient that needed transfusion. If safety is an endpoint in the study, please add that to the methods and results.

Safety is not an endpoint this study. We have already published a paper on the safety of weekly primaquine (Kheng et al. BMC Med 15, Taylor et al JID 2019). Nevertheless, we mention the changes in haemoglobin in relation to the changes in the reticulocyte count. For those readers who may not be familiar with our early results, we mention, en passant, the one blood transfusion.

Reviewer #3: Yes

--------------------

Reviewer #1: (No Response)

Reviewer #2: 8. AHA can also refers to autoimmune hemolytic anemia, so perhaps better not to use it as an abbreviation for acute hemolytic anemia.

We have changed this to acute haemolysis - AH.

9. If the goal of the study was to assess the performance of the test at baseline, then are the additional time points necessary?

Not entirely. The FST was only assessed at baseline. The other objective of the paper is to report the changes in G6PD activity and the reticulocyte count over time.

Reviewer #3: I'm not sure that very dense spaghetti plots are the best way to represent this data. I would favor mean or median over time for each group with error bars representing 95% CI or interquartile range. Box plots at each time point would also be much easier on the eyes.

See the comment above.
--------------------


Reviewer #1: The current study addressed two clinical questions: 1) whether diagnosis by FST is impacted by acute malarial infection in G6PDd males and G6PDhet females, and 2) how do reticulocyte dynamics change and influence G6PD activity during malaria infection/treatment in G6PDd and G6PDn individuals.

While the limitations of the first point are discussed below, it is worth mentioning that the retic and enzyme activity dynamics data and subsequent discussion are strong and quite compelling. This portion of the paper adds an important piece of information to the field, and is helpful for understanding the underlying retic/enzyme dynamics during acute malaria infection and treatment.

We thank this reviewer for crystalising these points.

To the first question, the authors claim that the evidence presented supports the notion that diagnosis by FST is not affected. Out of 75 total patients, 15 were G6PDd males and 3 G6PDhet females, and the FST correctly diagnosed all 15 males, and of the three females, two were classified as deficient while one was diagnosed as normal, and the authors cite “technical reasons” to explain a low reading on Day 0 and that it is more likely that she was ~5 U/ g Hb.

It would have been useful to include calculated sensitivities and specificities (both ~99-100%) as the major point of the study was to examine test characteristics in this population, and would have been even more helpful to include published test characteristics of FST in different G6PD variant populations, as well as the lower limit of detection of the FST test (if that data exist). That is, at what activity level (in U/ g Hb) does the FST give a qualitative diagnosis of G6PD deficiency.

We have estimated the sensitivity and specificity of the FST at baseline, cognizant that the numbers are small, and also brought out more previous work on the test characteristics of the FST and other qualitative tests.

Even if it is true that the single patient’s Day 0 activity is falsely low, it is an over-generalization to state that this study presents clear evidence that FST does not misdiagnose G6PD heterozygous females.

We have changed the tone of the interpretation of the FST. With only three heterozygous females, we cannot say much about the FST and we call for more research in this group (page 16, lines 271-273). However, the results are encouraging for G6PD deficient males.

Perhaps the authors should consider re-framing this conclusion. Furthermore, there seems to be a disconnect between the framing of the clinical problem in the Background section and the ensuing Discussion of results. Frankly, I was expecting the sample population to be more highly enriched for G6PD heterozygous females, and I am concerned that the target population was inappropriate to answer the proposed question.

The epidemiology of malaria in Cambodia is such that mostly young males are affected because they go to work in the forests. Therefore, the sample size composition is what we have. We agree that the number of heterozygotes is very small and this is a major limitation. This is why we are calling for more research in heterozygous females.

While this may be due to a possible heterozygous resistance to clinically-significant malaria infection or decreased access to treatment for Cambodian women, it is a significant limitation of the study that receives inadequate discussion. If it is not possible to enrich the sample with female heterozygotes due to a low volume seen at this clinic or for whatever reason, this would be helpful to mention while responding to these comments.

Yes, we agree with these comments and have amended the paper, accordingly.

Further, and with regard to the presentation of results, it would have been helpful to address the major clinical question earlier in the manuscript with the reticulocytes and G6PD connection occurring later.

Yes, we have reordered the results.

Regarding the reticulocyte and G6PD activity data, this was highly interesting and helpful to understand the increase in G6PD activity in the peri-infection/initiation of therapy period, though it is clear that the relationship between reticulocyte dynamics and G6PD activity is nuanced.

We thank this reviewer for this point.

More work will make the relationship clearer between G6PD activity and the reticulocyte count, especially in heterozygous females. In the paper, we cite work form the late 1950s that also shows a rising G6PD activity in parallel with a rising reticulocyte count in African American volunteers who were challenged with 30 mg of daily primaquine (Tarlov AIM 1962).

Specific Comments:
1. It would be helpful to designate which of the comparisons were non-parametric, and which assumptions specifically were violated. Furthermore, if the authors are significantly concerned about sample size, it might be helpful to display central tendencies as medians + 95% CIs, especially if the sample population is not normally distributed or sufficiently large enough to approach normality. In general, I would advocate for median w/ 95% CIs.

We have shown parametric and non-parametric analyses in Table 1.

We feel it is important to show ranges for the key clinical data – see above.

2. It would be helpful to include “A” and “B” designations for two-part figures, as well as titles for the y-axis. As it stands, no y-axis is labeled. Overall, the graphs cannot stand alone and can be difficult to interpret.

We have checked the graphs – all are labeled.

3. Which WHO classification does the Viangchan mutation belong to? Would we expect clinical manifestations at baseline and a corresponding reticulocytosis, or were the baseline retic counts expected? It would be helpful to illuminate these expectations for the reader.

Viangchan is a WHO class II variant, Canton is class III.

The point about baseline reticulocyte counts is well taken. However, there are, surprisingly, very few data on the reticulocyte count in vivax malaria. Limited data in P. falciparum suggest a tendency to lower reticulocyte counts.

4. The authors claim that G6PDd retics might have higher G6PD activity levels compared to G6PDn retics as evidenced by a higher relative increase in activity at Day 7 in G6PDd compared to G6PDn with a smaller increase in retic counts. Has this been reported previously or is there other evidence to support this claim?

It is known that reticulocyte counts have higher enzyme activity and we have included an additional reference in the paper (now 3 citations).

5. Is it known what is the activity level threshold to trigger hemolysis with the study anti-malarials?

Haemolysis is an inevitable consequence of using oxidant drugs like primaquine. Haemolysis is dose dependent and more severe in the more severe variants with lower enzyme activities. At low doses of primaquine, haemolysis is less in the so called milder G6PD variants but as the dose of primaquine increases, the degree of haemolysis becomes independent of variant.

6. Typically, the abbreviation “AHA” is reserved for “autoimmune hemolytic anemia,” not “acute hemolytic anemia.” I would re-consider this usage.

Yes, we have dropped this.

7. In Line 143, is this larger drop statistically significant? While it is stated that the antimalarial therapy was well-tolerated and only one patient required transfusion, it is clinically useful to recognize the hemolytic risks of these therapies.

Yes it is. We have added the p value, based on the D3-D0 changes in haemoglobin concentrations by G6PD status. .

8. In the regression models, did the authors adjust for confounding? If so, how was this dealt with statistically? Were any indicators not included in the full regression model?

Yes. They were multivariate analyses assessing the independence of each variable. We have now made this explicit by adding “independently associated” and “multivariable analysis” in the table 2 title.

9. Table 2 is difficult to understand. It is presumed that these are three reduced (final) models and the outcome variable is in bolded text. However, it is very unclear what “reticulocyte dynamics” means in the context of the model? What type of variable is it? How was it defined? What does it mean exactly? What predictor variables were included in the initial full models?

As above, we have dropped reticulocyte dynamics and revised the wording to “reticulocyte count over time”.

10. In Figure 3 title, please include the correlation coefficient for G6PDd.

We have done this.

11. It would be of major benefit to enrich the clinical sample with female heterozygotes and would substantiate the preliminary findings from just three female hets

See comment above on the epidemiology of malaria in Cambodia.

Reviewer #2: Summary: The authors measured the performance of a qualitative test for G6PD activity and found that it was accurate even in the setting of an active P vivax infection.

Reviewer #3: The authors have performed a study evaluating G6PD levels in subjects with acute vivax malaria and also its impact on the receipt of treatment. I have no concerns regarding the methodology, and to my knowledge this is novel. It asks and addresses a question that matters. The introduction and discussion are reasonable.

I only have one minor suggestion of relevance. I'm not sure that very dense spaghetti plots are the best way to represent this data. I would favor mean or median over time for each group with error bars representing 95% CI or interquartile range. Box plots at each time point would also be much easier on the eyes.

Again, we would like to keep the spaghetti plots for the reason explained above.
--------------------
